# Ovariectomy Results in Variable Changes in Nociception, Mood and Depression in Adult Female Rats

**DOI:** 10.1371/journal.pone.0094312

**Published:** 2014-04-07

**Authors:** Li-Hong Li, Zhe-Chen Wang, Jin Yu, Yu-Qiu Zhang

**Affiliations:** 1 Institute of Neurobiology, Institutes of Brain Science and State Key Laboratory of Medical Neurobiology, Fudan University, Shanghai, China; 2 Department of Integrative Medicine and Neurobiology, State Key Laboratory of Medical Neurobiology, Shanghai Medical Colloge, Fudan University, Shanghai, China; Peking University, China

## Abstract

Decline in the ovarian hormones with menopause may influence somatosensory, cognitive, and affective processing. The present study investigated whether hormonal depletion alters the nociceptive, depressive-like and learning behaviors in experimental rats after ovariectomy (OVX), a common method to deplete animals of their gonadal hormones. OVX rats developed thermal hyperalgesia in proximal and distal tail that was established 2 weeks after OVX and lasted the 7 weeks of the experiment. A robust mechanical allodynia was also occurred at 5 weeks after OVX. In the 5^th^ week after OVX, dilute formalin (5%)-induced nociceptive responses (such as elevating and licking or biting) during the second phase were significantly increased as compared to intact and sham-OVX females. However, chronic constriction injury (CCI) of the sciatic nerve-induced mechanical allodynia did not differ as hormonal status (e.g. OVX and ovarian intact). Using formalin-induced conditioned place avoidance (F-CPA), which is believed to reflect the pain-related negative emotion, we further found that OVX significantly attenuated F-CPA scores but did not alter electric foot-shock-induced CPA (S-CPA). In the open field and forced swimming test, there was an increase in depressive-like behaviors in OVX rats. There was no detectable impairment of spatial performance by Morris water maze task in OVX rats up to 5 weeks after surgery. Estrogen replacement retrieved OVX-induced nociceptive hypersensitivity and depressive-like behaviors. This is the first study to investigate the impacts of ovarian removal on nociceptive perception, negative emotion, depressive-like behaviors and spatial learning in adult female rats in a uniform and standard way.

## Introduction

Circulating ovarian hormones not only play a pivotal role in reproductive behavior and sexual differentiation, they also contribute to emotion, memory, neuronal survival and the perception of somatosensory stimuli [1]–[3]. Ovarian hormones have been shown to alter nociceptive behaviors using a variety of models [4]–[8]. However, the findings of these investigations have been variable. Depending on the type of noxious stimulation, behavioral test employed, species and strain of the animal and periods from ovariectomy (OVX), gonadectomy increased [4], [7], [9], decreased [10], [11] or had no effect [12] on nociceptive responses.

Ovarian hormones have also been suggested to regulate affective disorders and learning memory beyond their role in pain modulation [3]. Human studies have demonstrated that the lifetime prevalence rate of mood disorders is approximately two times more frequent in women than in men [13], [14]. The increased risk of affective disorders in women is related to hormonal changes who are premenstrual, postpartum and hypoestrogenic due to the medical surgery or menopause [15]. Data from animal experiments have also shown that OVX increases depressive-like behavior in several tasks [16], [17]. In addition, very low estradiol levels or very high levels were associated with impaired spatial ability [18], [19].

In humans, menopause causes depletion of estrogens, whereas in experimental animals OVX is a common method to deplete animals of their gonadal hormones. In females the absence of the ovaries induces a drastic decrease of circulating estrogens [20]. Our previous studies demonstrated that exogenous 17β-estradiol acutely enhanced excitatory synaptic transmission in spinal dorsal horn and anterior cingulate cortex (ACC) neurons and facilitated nociceptive responses and pain-related aversive in intact rats either sex [21], [22]. In this study, we are further to compare intact female rats to OVX ones to determine if hormonal depletion could significantly modify the nociceptive, depressive-like and learning behaviors under controlled experimental conditions. The Tail-flick test was used to determine the thermal pain threshold; the Von Frey test was used to measure the mechanical response threshold; formalin test was used to examine tonic pain. Chronic constriction injury (CCI) of the sciatic nerve was used to induce neuropathic pain. Formalin-induced conditioned place aversion (F-CPA) and foot-shock-induced CPA (S-CPA) was respectively used to reflex the pain-related and fear-related aversion. Force swimming test, and open-filed test (OFT) were used to evaluate depressive-like behaviors [23]. Morris water maze was used to test spatial learning ability.

## Materials and Methods

### Experimental Animals

Adult female Sprague Dawley rats (weighting 200–220 g on arrival, from the Experimental Animal Center of the Chinese Academy of Sciences were housed in groups in a temperature- and humidity-controlled room with a 12∶12 light-dark cycle (lights on 06∶00) and with food and water available *ad libitum*. All animal experiments were approved by the Shanghai Animal Care and Use Committee and followed the policies issued by the International Association for the Study of Pain on the use of laboratory animal [24]. To control for possible effects of time of day, rats were trained and tested at approximately the same time of day (3–6 h after lights on). Gonadally intact females were randomly cycling. All the following behavioral testing described herein were performed by the same experimenter blinded to the group assignment to minimize between-experimenter.

### Surgery

Ovariectomies (OVX) were performed under isoflurane anesthesia. Anesthesia was confirmed by a reduced respiratory rate and lack of response to gentle pinching of the foot pad. A mid-ventral incision was made, and the bilateral ovaries and ovarian fat were removed. The ovaries were isolated by ligation of the most proximal portion of the oviduct before removal. The same procedure was carried out for the sham groups except for the removal of the ovaries. The surgical incision was sutured and postsurgical recuperation was monitored daily.

Chronic constriction injury (CCI) of the sciatic nerve was performed according to previous protocols [25]. The right sciatic nerve was exposed at the mid-thigh level, and four chromic gut (4-0) ligatures were tied loosely around the nerve approximately 1 mm apart, proximal to its trifurcation. For sham group rats, the sciatic nerve was isolated without ligation. The surgical incision was sutured and postsurgical recuperation was monitored daily.

### Oestrous cycle evaluation

Stages of the oestrous cycle (diestrus, proestrus, oestrus and metestrus) in female rats [26] were determined using vaginal smears collected about 2 h after lights on every day. Cycles were followed for at least 2 weeks before animal treatment. Only those who maintained at least two consecutive 4- or 5-day oestrous cycles were considered to be regulatory cycling and were used in the present study.

### Plasma estrogen concentration and weight of body and uterus

Rats were anesthetized with isoflurane and blood samples were collected by femoral artery puncture and put into tubes pretreated with heparin. The samples were let stand for one hour before centrifuging at 14000 rpm for 10 minutes. The blood serum was then collected and stored at −80°C until use. Serum estradiol levels were determined by double-antibody radioimmunoassay kits according to protocols provided by the manufacturer (National Atomic Energy Research Institute, Beijing, China).

The body weight of each animal was recorded every week throughout the experiment and the uterus weight was measured postmortem at the end of the 5^th^ week as indices of the efficiency of the OVX.

### Estrogen replacement

Estrogen replacement was carried out in the OVX rat 4 week after surgery and by subcutaneous (s.c.) injection of 17β-estradiol (E2, 30 μg/day for 7 days) in the dorsal neck region. The same volume of vehicle (sesame oil) was injected into OVX rats daily as a control. Behavioral tests were carried out in the 1 week after E2/vehicle treatment.

### Von Frey test

The hind paw withdrawal threshold (PWT) was determined using a calibrated series of Von Frey hairs (Stoelting, IL, USA) ranging from 2 to 26 g. Rats were placed individually into wire mesh-bottom cages. A series of calibrated Von Frey hairs were applied to the plantar surface of the hindpaw in ascending order (2, 4, 6, 8, 10, 15, and 26 g) with a sufficient force to bend the hair for 2 s or until paw withdrawal. A withdrawal response was considered valid only if the hindpaw was completely removed from the customized platform. Each hair was applied 5 times and the minimal value that caused at least 3 responses was recorded as the PWT.

### Tail Flick Tests

Rats were gently handled on the platform of tail flick testing apparatus (BME-410C, Institute of Biomedical Engineering, CAMS, Tianjin, China) with tails exposed to thermal stimuli. Radiant heat was applied to the proximal (3.0 cm from the root) or distal (3.0 cm from the tip) of the rat's tail to evoke the tail-flick (TF) reflex. When a “flick” reaction occurs, an on-board sensor turns off the bulb, the second counter is stopped, and the tail-flick latency is determined to the nearest 0.01 sec. The intensity of radiant heat was adjusted to elicit a baseline TF latency (TFL) of approximately 4–5 s. Cutoff time was 10 s to avoid tissue damage.

Von Frey and Tail Flick tests were performed in the same groups of OVX/sham/naïve rats, started with Von Frey test followed by TFL test with an interval of 2 hours in home cages.

### Formalin Test

Formalin (5% 50 μL) was injected into i.pl. of the unilateral hindpaw. The lifting and licking time of the affected paw during each 5 min interval for 45 min after injection was recorded. A mirror was positioned below a chamber at a 45° angle for unobstructed observation of the rat's paws. The responses to formalin injection were manually monitored by measuring the time the animal spent on lifting, licking, and shaking the affected paw. The behaviors of each animal were simultaneously monitored by a video camera. A weighted pain score for each animal was calculated using the following formula[27]: formalin pain score  =  [the time the animal spent on elevating injected paw+2× (the time the animal spent on licking or biting injected paw)]/300. The different groups of OVX/sham/naïve rats were used for this test.

### Conditioned place aversion

Conditioned place aversion (CPA) was conducted as described previously [28]. The place conditioning apparatus consists of three opaque acrylic compartments. Two large ones are conditioning compartments (30×30×30 cm) and a smaller one is a neutral choice compartment (15×20×30 cm, length x width x height). The conditioning compartments are placed in parallel and separated by a wall with a square door (10×10 cm). The neutral compartment is laid in front of the two conditioning compartments with two doors (10×10 cm) to them. A movable transparent ceiling covers each compartment. The two conditioning compartments are both painted black, but one is decorated with a transverse white band and contains an odor produced by 1.0% aceticacid; the other is decorated with a white vertical band and has an odor of cinnamon. The floors of the conditioning compartments are also different: one is made from Plexiglas, and the other is from a polyester board with a metal band on it, which can provide an electric shock. Thus, the two conditioning compartments have distinct visual, olfactory and tactile cues. The neutral compartment is white, absent of distinctive odor and has a solid acrylic floor with a slope. Under each of the floors of the conditioning compartments, there was a spring balance. When the animal stepped on the floor, it induced the movements of the balance. The mechanical energy was transformed into electrical signals. The signal trigged a timer, which automatically recorded the time spent in this compartment by the animal.

The experimental process consists of three distinct sessions: a preconditioning session, a conditioning session and a test (postconditioning) session. CPA task processing takes 4 d. Day 1 is a preconditioning day. At the beginning, a rat was placed in the neutral compartment. After habituating for 2 min, the entrance to each conditioning compartment was opened. When the rat enters any conditioning compartment, the doors connecting neutral and conditioning compartments were closed. The rat was allowed to explore the two conditioning compartments freely for 15 min. A timer automatically recorded the time spent in each of the compartments in a blind manner. Rats that spent >80% (720 s) on one side on that day were eliminated from the subsequent experiments [27], [28]. Day 2 and 3 are conditioning days. For formalin-induced CPA (F-CPA) experiment, the rat received a unilateral hindpaw intraplantar injection of normal saline (NS, 50 μl) on day 2, and was randomly confined to one of the conditioning compartments for 45 min. On day 3, the rat was given a unilateral hindpaw intraplantar injection of 5% formalin (50 μl) or NS (control) and then restrained in the other conditioning compartment for 45 min. For electric foot-shock-induced CPA (S-CPA) experiment, the rat received no treatment on day 2, and was randomly confined to one of the conditioning compartments for 45 min. On day 3, the rat received an electric shock (0.5 mA for 2 s) every 8–10 min in the other conditioning compartment during the 45-min training session. The compartments were counterbalanced. Day 4 is postconditioning day. The procedure is the same as day 1. The time animals spent in each compartment was measured. The different groups of OVX/sham/naïve rats were used for F-CPA and S-CPA tests.

### Open-field test

The open field apparatus consisted of a grey Plexiglas quadratic box (100×100×40 cm), which was evenly illuminated to 15 lux. The bottom of the box was divided into 16 squares. Each rat was placed in the center of the apparatus and allowed to explore the field for 3 min. During the test time, the number of crossings (defined as at least three paws in a quadrant) and rearing (defined as the animal standing upright on its hind legs) were measured. The behaviors of each animal were simultaneously monitored by a video camera. The test apparatus was cleaned with a 10% ethanol solution and water to remove any olfactory cues between animals.

### Forced swimming test

The design of the forced swimming test was adapted from previous description [29]. Briefly, rats were forced to swim individually in a cylindrical glass container (40 cm height, 18 cm diameter), which contained tap water (24±2°C) to a depth adjusted for the weight of the individual animal, so that its hind paws could just touch the bottom of the container. After 15 min in the water, rats were removed and allowed to dry for 15 min in a heated container before being returned to their home cages. Rats were replaced in the cylinders 24 h later and total duration of immobility (immobility time) and activity (activity time) was measured during a 5-min test. The behaviors of each animal were simultaneously monitored by a video camera. Immobility was defined as rat not making any active movements other than that necessary to keep the head and nose above the water (e.g., when rats were floating in a vertical position). Activity included climbing (presenting active movements with the forepaws in and out of the water, usually directed against the walls) and swimming (showing active swimming motions, more than those necessary to keep the head above water, i.e. moving around in the cylinder or diving).

Open-field and forced swimming tests were performed during two consecutive days in the same groups of OVX/sham/naïve rats. Day 1 started with open-field test (OFT) followed by 15-min forced swimming training with a rest for 2 hours in home cages. On Day 2, forced swimming test was performed.

### Morris water maze task

The water maze consists of a black round tank, which has a diameter of 150 cm and height of 54 cm and is filled with water (24±2°C) to a depth of 38 cm. The water is made opaque by black food coloring so that the submerged platform (9.0 cm in diameter, 2.0 cm below the water surface) is invisible. Fixed, extra-maze visual cues were present at various locations around the maze (i.e., computer, video camera, posters) in the room with constant brightness (25 lux). The training and testing protocols were essentially as described [28]. The training procedure consists of two sessions with a 30 min interval in between, each session consisting of six consecutive trials. The submerged platform is located at the central position of the southeast quadrant of the tank. The starting position is randomly selected, but counterbalanced among the four positions. A rat was allowed to search for the submerged platform for 60 s. If successful in locating the platform within 60 s, the rat was allowed to stay on the platform for 30 s; if not, it was directed to the platform and allowed to stay there for 30 s. Thereafter, the rat was returned to a holding cage. The next trial began after an intertrial interval of 30 s.

A three-trial retention test was conducted 24 h after the training. For each rat at each trial, the submerged platform was fixed at the target quadrant and the starting point was at the position opposite to it. Each rat was giving 60 s to locate the submerged platform. If successful in finding the platform within 60 s, the rat was immediately returned to a holding cage for 60 s before next trial began. If unsuccessful in locating the platform within 60 s, the rat was directed to the platform and allowed to stay there for 30 s, and then was returned to a holding cage for 30 s before the next trial.

Immediately after the retention test was completed, the rat was tested in a visible platform version of the Morris water maze. The platform was raised to above the water surface and covered with white gauze to make it highly visible. Each animal was placed on the visible platform for 30 s before testing. The starting position for any given rat from the groups was selected randomly, but once selected it was fixed for that rat, whereas the visible platform was randomly placed among the four quadrants. The rat was allowed to locate the visible platform for 60 s in each trial. If successful in finding the platform, the rat was returned immediately to a holding cage; if not, the rat was removed from water and returned to a holding cage. The next trial began after an intertrial interval of 60 s. A total of three trials were conducted for each rat. Navigation of each animal in the water maze was monitored using a video camera, a tracking system and tracking software (Ploly-Track Video Tracking System, San Diego Instruments). Using the tracking software, escape latency, and swimming traces and speed were recorded for subsequent analysis.

### Statistical analysis

Data are presented as mean ± SEM. Student's t-test, paired t-tests, one-way, two-way ANOVA or repeated measures ANOVA (RM ANOVA) followed by post hoc Student–Newmann–Keuls test were used to identify significant differences. In all cases, P<0.05 was considered statistically significant.

## Results

### Mechanical response thresholds and thermal pain thresholds were not affected by estrous cycle

**Figure 1:**
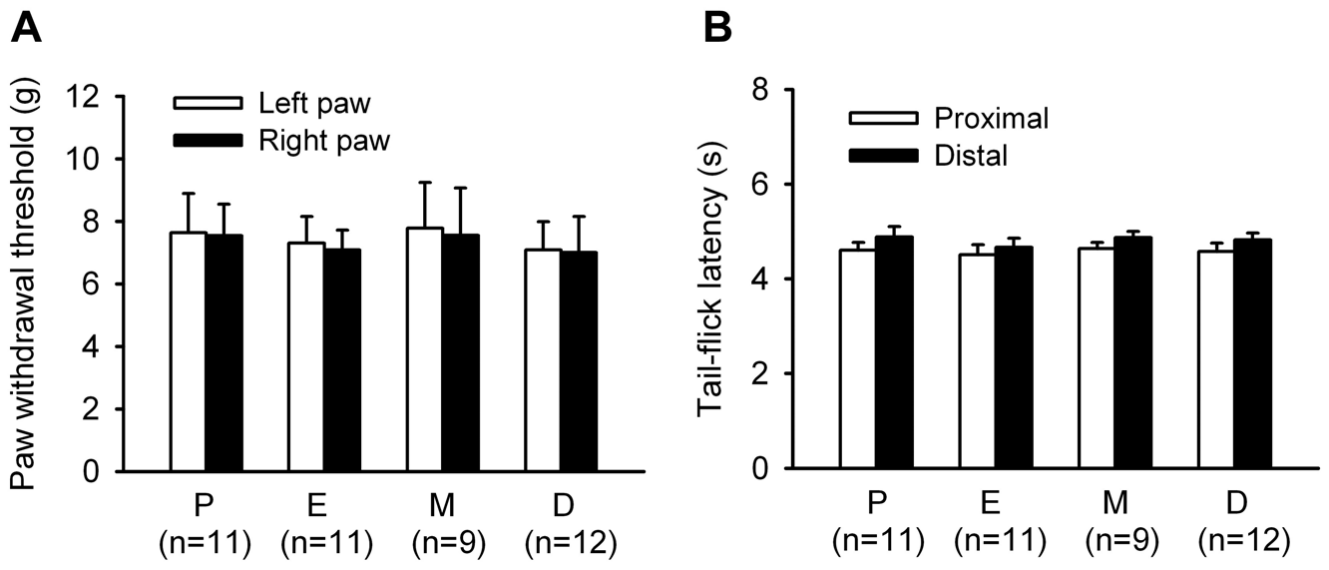
Effects of estrous cycle on basal paw withdrawal threshould (PWT) to mechanical stimulation and tail-flick latency (TFL) to radiant heat stimulation. (A) PWTs of both hindpaws. (B) TFLs of proximal and distal. P, proestrus stage; E, Estrus stage; M, metaestrus stage; D, diestrus stage.

Intact female rats were primarily classified as in proestrus, estrus, metestrus or diestrus on the day of testing according to the cellular characteristics of their vaginal smears. All rats had 4–5 day cycles. Baseline measures of paw withdrawal thresholds (PWTs) to von Frey hairs on both hindpaws did not differ across estrous phases (One-way ANOVA, Left: F_ 3,39_ = 0.0916, p = 0.964; Right: F_3,39_ = 0.0732, p = 0.974) (Fig. 1A). Radiant heat-evoked TF reflexes were measured in proximal and distal of the rat's tail with a time interval of 15 min between tests. Similar to PWTs, neither proximal nor distal tail-flick latencies (TFLs) was altered by estrous cycling (One-way ANOVA, distal: F_3,39_ = 0.323, p = 0.809; proximal: F_3,39_ = 0.108, p = 0.955) (Fig. 1B).

### Ovariectomy induced mechanical allodynia and thermal hyperalgesia

**Figure 2:**
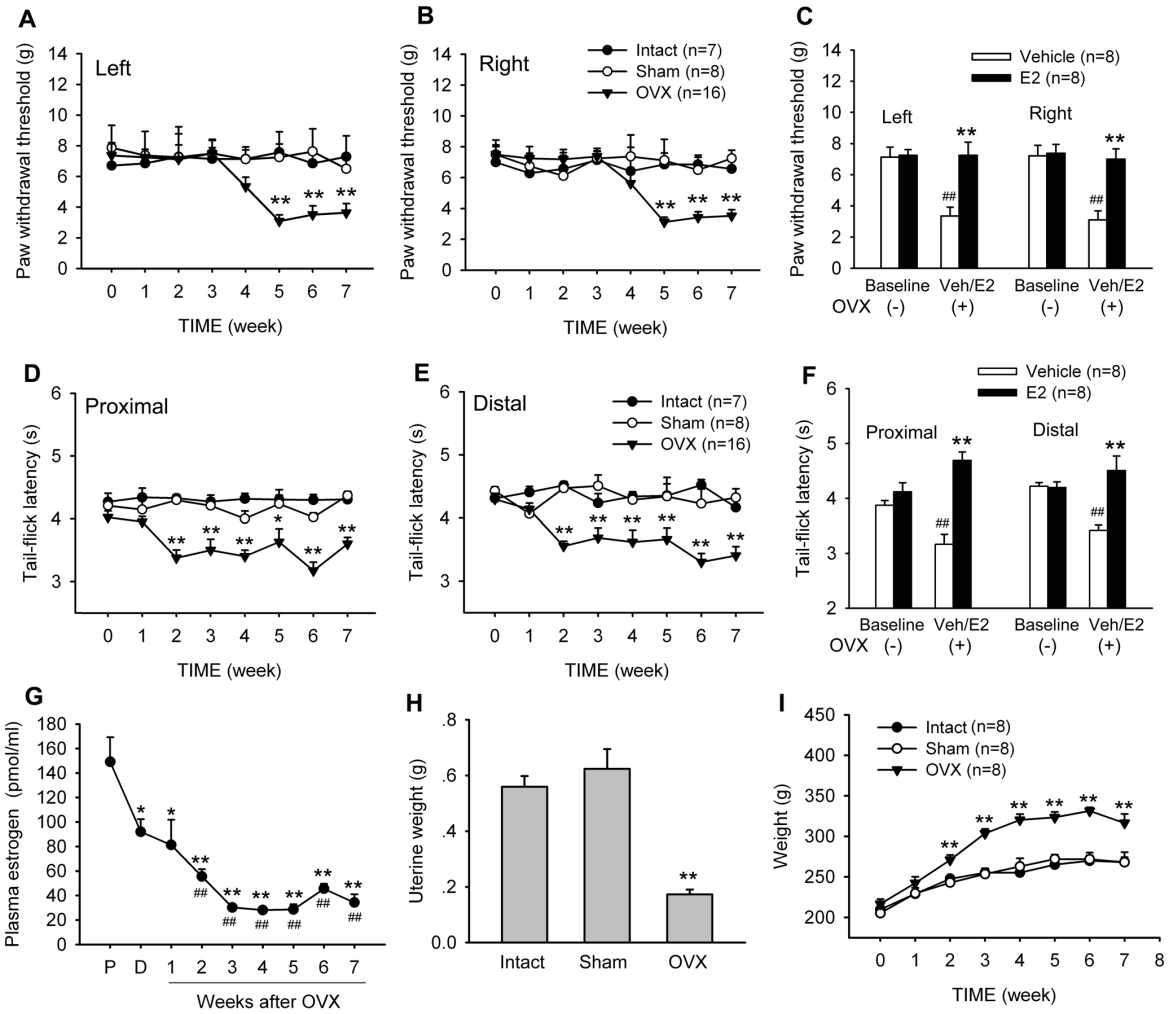
OVX induced mechanical allodynia and thermal hyperalgesia. (A & B) Effects of OVX on PWTs of both hindpaws. ** p<0.01 versus sham-OVX and intact. (C) Effects of 17β-estradiol (E2) or aequales vehicle (sesame oil) on PWTs of OVX rats. ** p<0.01 versus vehicle control; ## p<0.01 versus baseline. (D & E) Effects of OVX on TFLs of proximal and distal. * p<0.05; ** p<0.01 versus sham-OVX and intact. (F) Effects of E2 or aequales vehicle on TFLs of OVX rats. ** p<0.01 versus vehicle control; ## p<0.01 versus baseline. E2 (30 μg) was subcutaneously injected into OVX rats daily for 7 days from the 4^th^ week after OVX surgery. Behavioral tests were performed at the 5^th^ week. (F) Plasma estrogen concentration was significantly decreased after OVX. * p<0.05, ** p<0.01 versus proestrus stage; ^##^ p<0.01 versus diestrus and proestrus stage. (G) The uterine tube weight was attenuated at the 5^th^ week after OVX. (H) The body weight was significantly increased after OVX. ** p<0.01 versus sham-OVX and intact. P, proestrus stage; D, diestrus stage.

In the absence of ovariectomy (OVX), PWTs to von Frey hairs were stable for at least 7 weeks. Following OVX, PWT values significantly lower in 5–7 weeks after surgery as compared with that of intact and sham OVX rats, suggesting that OVX rats developed a mechanical allodynia (Fig. 2A and 2B). Two-way RM ANOVA analysis revealed significant effect of treatment groups (Left: F_2,29_ = 11.233, p = 0.002; Right: F_2,29_ = 8.668, p = 0.005). In E2-treated OVX rats (s.c. 30 μg/day for 7 days from the 4^th^ week after OVX), PWTs were significantly higher than that of vehicle (sesame oil)-treated ones at the 5th week after OVX (Student's t-test, Left: t_0.01,7_ = 3.844, p<0.01; Right: t_0.01,7_ = 4.449, p<0.01) (Fig. 2C).

A significant thermal hyperalgesia also developed from 2 weeks after OVX and lasted for more than 7 weeks in tail-flick reflex tests (Fig. 2D and 2E). Two-way RM ANOVA analysis revealed significant effect of OVX (Proximal: F_2,29_ = 53.002, p<0.001; Distal: F_2,29_ = 24.646, p<0.001) and significant interaction between groups and time (Proximal: F_14,203_ = 3.628, p<0.001; Distal: F_14,203_ = 6.556, p<0.001). Similar to OVX-induced allodynia, thermal hyperalgesia was also reversed by E2 replacement for 7 days (Student's t-test, Proximal: t_0.01,7_ = 5.702, p<0.01; Distal: t_0.01,7_ = 3.462, p<0.01) (Fig. 2F).

Surgical ovariectomies were confirmed by measure of plasma estrogen concentration, uterus weight and vaginal smears. After OVX, regular 4-day estrus cycle disappeared within one week and the plasma estrogen concentration decreased significantly at all the time-points we examined (One-way RM ANOVA, F_8,63_ = 10.676, p<0.001) (Fig. 2G). In addition, consistent with previous reports [30], OVX caused a significant decrease in uterus size and increase in body weight (Fig. 2H and 2I).

### Ovariectomy enhanced formalin-induced tonic pain but did not affect nerve injury-induced neuropathic pain

**Figure 3:**
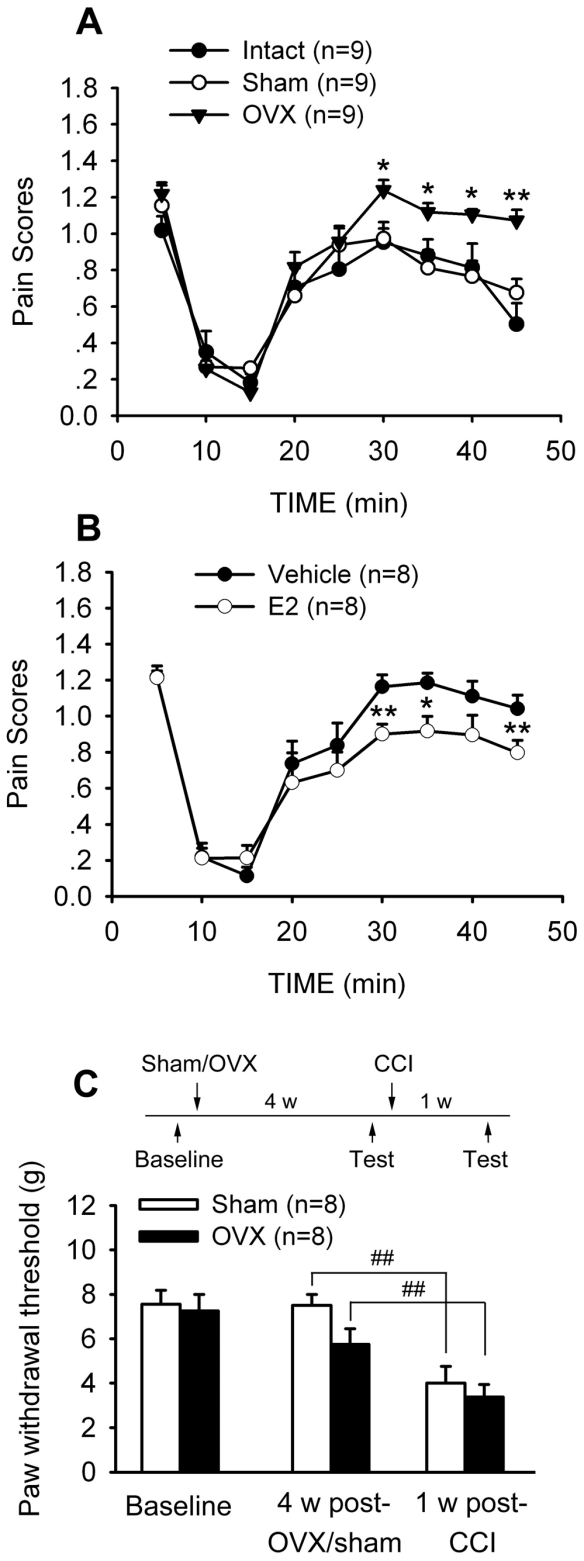
OVX enhanced formalin-induced nociceptive responses during phase 2 but did not affect CCI-induced mechanical allodynia. (A) OVX rats showed a higher formalin pain scores during phase 2. * p<0.05, ** p<0.01 versus sham. (B) Subcutaneous injection of E2 (30 μg/day for 7 days) significantly suppressed formalin pain scores during phase 2. * p<0.05, ** p<0.01 versus vehicle control. E2 and vehicle were injected from the 4^th^ week after OVX, and formalin test was performed at the 5^th^ week. (C) Both sham-OVX and OVX groups developed mechanical allodynia on day 7 after CCI. There was no significant difference in PWTs between sham and OVX rats. CCI surgery was performed at 4 weeks after sham-OVX or OVX. ^##^ p<0.01.

Intraplantar (i.pl.) injection of 5% formalin (50 μL) into unilateral hindpaw produced typically two phases of nociceptive behavioral response including licking, shaking, elevating and clutching and favoring the affected paw. An early response (phase 1) lasted about 5 min followed by a 5–10 min period of decreased activity, and then, a late response (phase 2) lasted about 40 min. As shown in Fig. 3A, OVX rats showed higher pain scores than intact and sham rats. Two-way RM ANOVA analysis revealed the significance of the factor OVX (F_2,24_ = 6.252, p = 0.001). In E2-treated OVX rats, formalin pain scores were significantly decreased as compared to vehicle controls (Two-way RM ANOVA, F_1,14_ = 8.402, p<0.01) (Fig. 3B).

Surgery for chronic constriction injury (CCI) of the sciatic nerve was performed at 4 weeks after OVX or sham-OVX. Strong mechanical allodynia developed in the ipsilateral hindpaw on day 5 either in OVX or sham-OVX rats (Fig. 3C). No statistical difference in the PWTs was found between OVX and sham-OVX groups.

### Ovariectomy attenuated pain-related aversion but did not affect fear-related aversion

**Figure 4:**
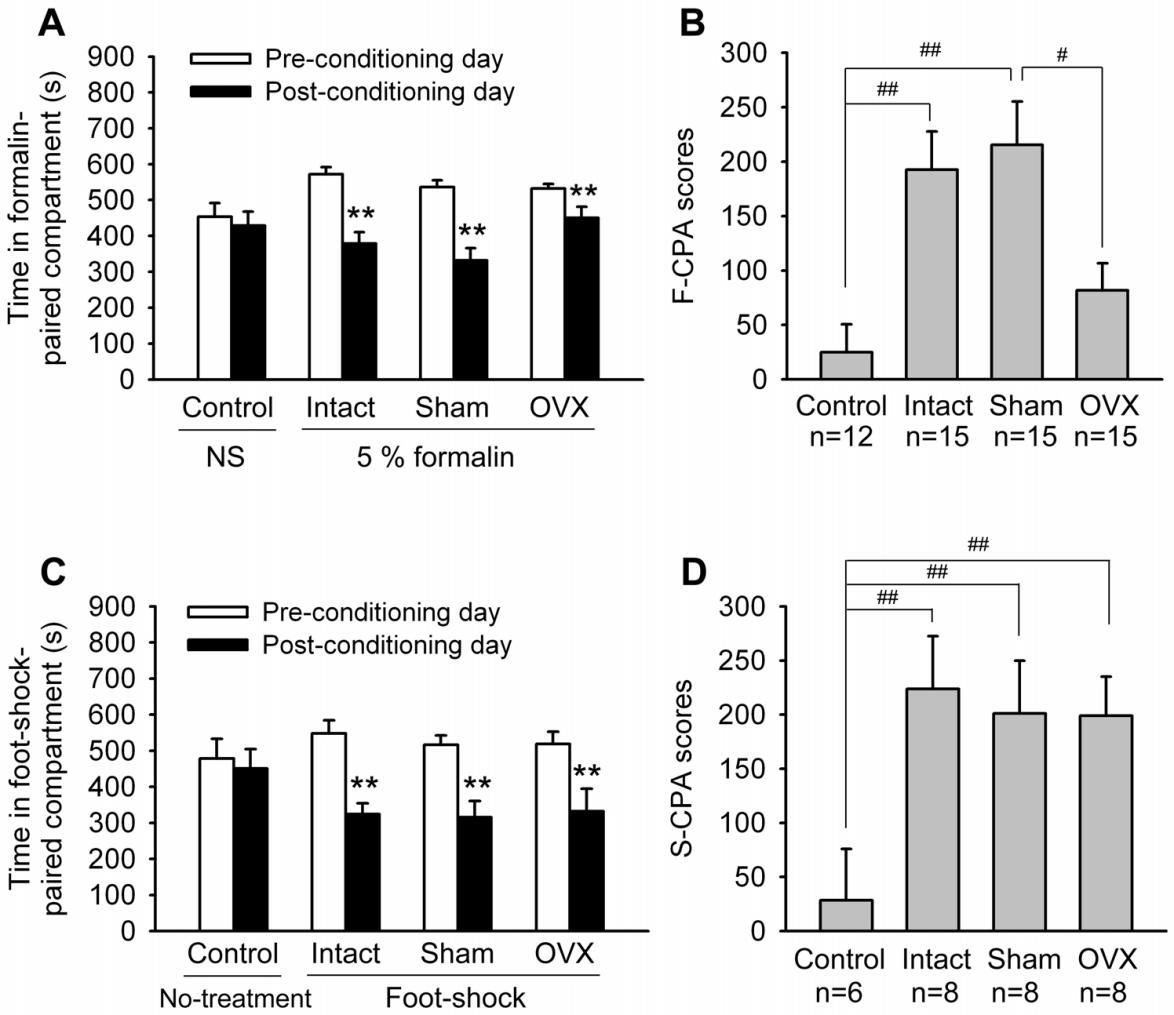
OVX suppressed F-CPA but did not affect S-CPA. (A & B) Effects of OVX on formalin-induced CPA (F-CPA), as indicated by time spent in the treatment (formalin or normal saline)-paired compartment on preconditioning and postconditioning days (A) and CPA scores (the time spent in the treatment-paired compartment on the pre-conditioning day minus that on the post-conditioning day (B). (C & D) Effects of OVX on foot shock-induced CPA (S-CPA), as indicated by time spent in foot shock-paired compartment on preconditioning and postconditioning days (C) and CPA scores (D). ** p<0.01 versus preconditioning day. ^#^ p<0.05, ^##^ p<0.01.

Intradermal injection of diluted formalin is painful in human[31]. The fact that formalin injection produces both conditioned place aversion (CPA) and other nociceptive behaviors in animals indicates that formalin is aversive to animals in a manner resembling the response to noxious stimuli in humans. Thus, formalin-induced CPA (F-CPA) is believed to reflect the emotional component of pain [32]. When a unilateral i.pl. injection of formalin (5%, 50 μL) was paired with a particular compartment in the place conditioning apparatus, all the intact, sham-OVX and OVX rats spent less time in this compartment on the post-conditioning day compared with the pre-conditioning day (Paired t-test, intact: t_0.01,14_ = 5.517, P<0.001; sham-OVX: t_0.01,14_ = 5.488, P<0.001; OVX: t_0.01,14_ = 3.701, p = 0.002). Control animals with an i.pl. injection of NS did not exhibit CPA (Fig. 4A). However, the CPA score [the time spent in the treatment paired compartment on the pre-conditioning day minus the time spent in the same conditioning compartment on the post-conditioning day] in OVX group was significantly lower than that in intact and sham-OVX groups (Fig. 4B) (One-way ANOVA, F_2,42_ = 3.884, P = 0.028), implying that ovarian hormones in adult female rats may be involved in pain-related negative emotion.

The electric foot-shock at the low intensity of current (0.4–0.8 mA) is routinely used as a fear conditioning. When an electric foot-shock (0.5 mA) was paired with a particular compartment in the place-conditioning apparatus, the rats spent significantly less time in this compartment on the post-conditioning test day as compared with the preconditioning test day (Fig. 4C). The CPA scores were not significantly different between OVX and control (intact and sham-OVX rats) groups (Fig. 4D) (One-way ANOVA, F_2,21_ = 0.0776, P = 0.926), suggesting that ovarian removal did not affect fear-related aversion.

### Ovariectomy increased depressive-like behaviors but did not impair spatial performance in the Morris water maze

**Figure 5:**
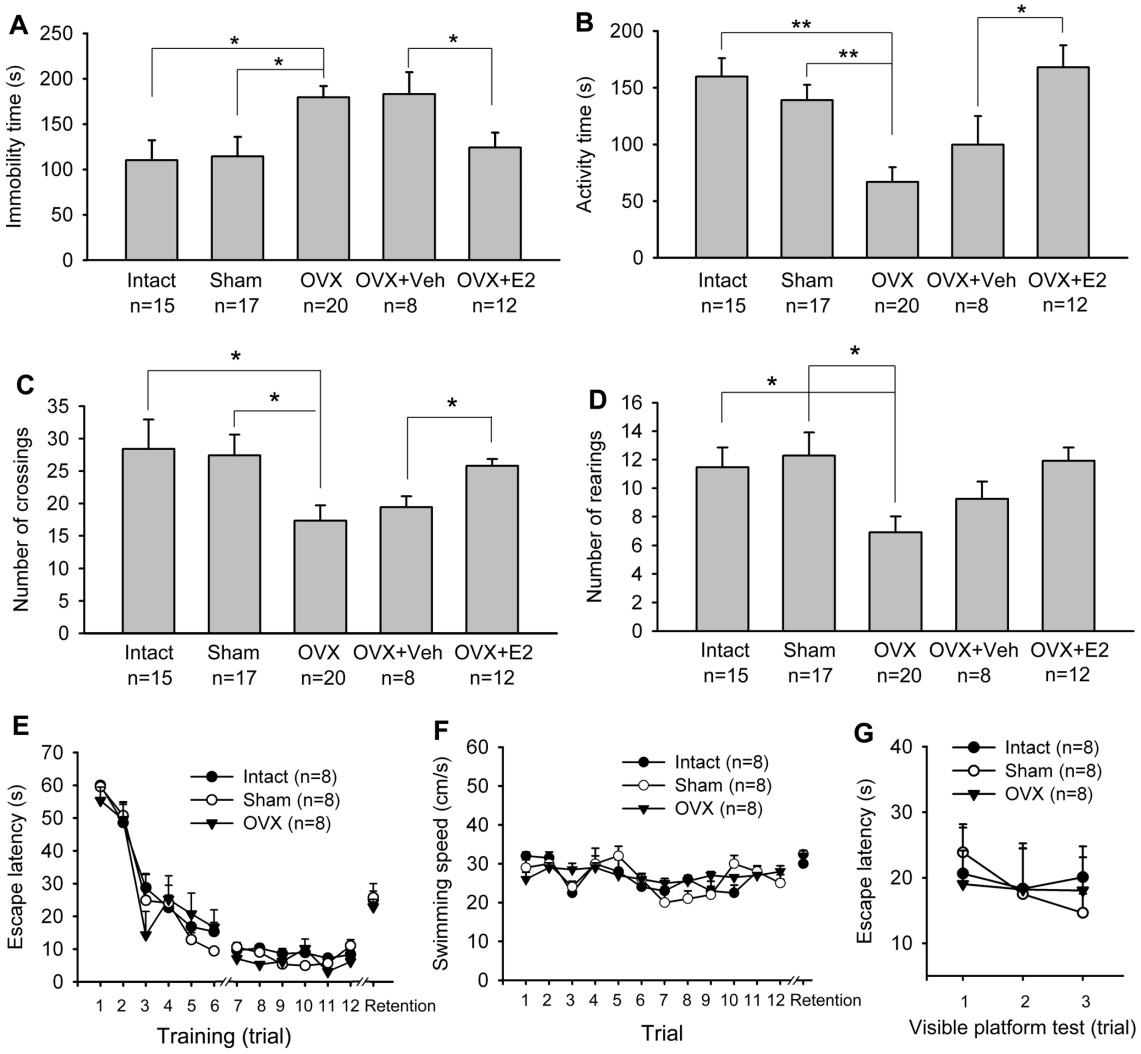
Effects of OVX on depressive-like behaviors and spatial ability. (A & B) OVX rats displayed significantly more immobility (A) and less activity (B) time in FST. Subcutaneous injection of E2 (30 μg/day for 7 days) significantly attenuated immobility and prolonged activity time. * p<0.05, ** p<0.01. (C & D) OVX rats displayed fewer crossing (C) and rearing (D) numbers in OFT. E2 replacement increased crossing and rearing numbers in OFT. * p<0.05, ** p<0.01. E2 and vehicle were injected from the 4^th^ week after OVX, and behavioral tests were performed at the 5^th^ week. (E) Showing animal's escape latencies to find the submerged platform. Cutoff time was 60 s. (F) Showing animal's swimming speed in the training and testing trials. Morris water maze training consists of two training sessions of six trials each with a 30-min resting period between the two sessions. Memory retention was tested 24 h after training. The retention values are calculated as the mean of three-trial retention test. (G) Animal's escape latencies to find the visible platform. The platform was raised above the turbid liquid surface to be visible. FST, OFT and Morris water maze tests were performed at the 5^th^ week after OVX or sham-OVX surgery.

The forced swimming test was used to evaluate behavioral despair. Depressive-like behavior (behavioral despair) was defined as an increase in the time (in seconds) spent immobile. At the 5th week after OVX, rats showed a significant increase in immobility time and decrease in activity time as compared to sham-OVX and intact rats (One-way ANOVA, Immobility: F_2,49_ = 4.771, P = 0.013; Activity: F_2,49_ = 9.954, P<0.001). Following E2-treated (s.c. 30 μg/day for 7 days from the 4th week after OVX), rats showed a decreased immobility and increased activity time at the 5^th^ week as compared to vehicle-treated OVX (Student's t-test, Immobility: t_0.05,18_ = 2.728; Activity: t_0.05,18_ = 2.730) (Fig. 5A and 5B).

The open-field test (OFT) was use to determine locomotion and exploratory behavior. At the 5th week after OVX, rats showed significant decrease in the number of crossings and rearings as compared to sham-OVX and intact rats (One-way ANOVA, Crossings: F_2,49_ = 3.264, P = 0.047; Rearings: F_2,49_ = 4.828, P = 0.012). Similar change was also seen in the vertical activity(One-way ANOVA,). Following E2 replacement, the number of crossings was significantly increased as compared to vehicle-treated OVX rats. (Student's t-test, t_0.05,18_ = 2.327). E2 treatment also increased the number of rearings of OVX rats, although this increase in rearings did not reach statistical significance (Fig. 5C and 5D).

At the 5^th^ week after OVX or sham-OVX surgery, rats were run in the Morris water maze. As shown in Figure 5E, three groups of rats including intact and received OVX or sham-OVX surgery were able to learn to find the submerged platform in the Morris water maze; the escape latencies became shorter with increased numbers of training trials in all groups (Two-way RM ANOVA, groups: F_2,21_ = 0.697, p = 0.514; group x trials: F_22,231_ = 0.828, p = 0.688). Moreover, when memory retention was tested 24 h after training, the OVX rats also displayed escape latencies that were similar to those of the intact and sham-OVX rats (Fig. 5E). To evaluate visual ability of the rats, a visible platform version of the test was performed. The three groups showed no distinction in performance in visible platform test, nor in swimming speed in the training and retention trials (Fig. 5F and 5G).

## Discussion

Numerous studies on both animals and human subjects have demonstrated the potential effects of ovarian hormones on pain transmission, emotion, learning and memory, but the literature is not unanimous [1], [3], [14]. In the present study, we observed the impacts of ovarian removal on nociception, negative emotion and spatial learning memory simultaneously in the same experimental conditions, providing a series of normalized behavioral data.

### Ovariectomy and pain

One of the main observations of our study is that ovariectomy (OVX) of adult female rats induced a robust nociceptive hypersensitivity characterized by mechanical allodynia in hindpaws and thermal hyperalgesia in proximal and distal tail, as well as enhanced formalin pain scores. We were unable to detect significant differences in the nerve injury-induced mechanical allodynia between OVX and sham-OVX using the CCI model. Following unilateral CCI, all intact, sham-OVX and OVX rats developed mechanical allodynia within 5 days without differences in paw withdrawal thresholds (PWTs) of the three groups, which is consistent with a previous study [8]. We have also observed no estrous cycle differences in PWTs and tail-flick latencies (TFLs) in normal animals, confirming Sanoja and Cervero' s study in adult female mice [7].

Although there are studies reporting no changes or decreases in sensitivity to nociceptive stimuli following OVX [10], [12], there are many more studies demonstrating somatic and visceral hyperalgesia in OVXs. For example, Sanoja and Cervero [7], [33] described that OVX induced an increased visceral sensitivity and robust mechanical allodynia and hyperalgesia in the abdomen, hindlimbs and tail in mice. Bradshaw and Berkley [34] observed variable amounts of vaginal hyperalgesia measured by escape responses to vaginal distension and Ceccarelli et al.[4] reported that OVX rats showed longer pain responses to the formalin test. In the present study, we further showed that estrogen replacement for 1 week significantly reversed OVX-induce nociceptive hypersensitivity, suggesting that supplementary of estrogen produces antinociception in OVX animals. Consistently, previous studies have demonstrated that estradiol significantly decreased formalin-induced nociceptive responses in OVX rats [35]–[37]. Also, the antinociceptive effect of estradiol on adjuvant-induced hyperalgesia was reported [38]. Very recently, a study of Yan and colleagues indicated that estrogen can trigger vagus-mediated antinociception in a rat model of visceral pain [39]. Taken together, a decrease in plasma estrogen level may parallel an increase in nociceptive sensitivity, while elevated estrogen level may antagonize ovarian hormones depletion-induced pain facilitation.

Intriguingly, we observed a significant thermal hyperalgesia in tail at the 2^nd^ week lasting for 7 weeks after OVX, paralleled to the time course of decrease in plasma estrogen levels. However, mechanical allodynia in hindpaws took 5 weeks to develop, implying differences in the effects of endogenous ovarian hormones on mechanical and thermal nociceptive processing. Differently, Ma et al. observed that robust decrease in PWTs in Von Frey test appeared at the second week and persisted for at least 6 weeks after OVX without associated thermal hyperalgesia [9]. While a study from Chen et al. showed that both mechanical allodynia and thermal hyperalgesia were detected from the first to 8^th^ week after OVX [40]. It is worth noticing that heat thresholds may be influenced by skin temperature. It has been reported that OVX significantly elevated tail skin temperature in rats [41]. In the present study, it could not be rule out that elevated tail skin temperature might contribute thermal hyperalgesia of OVX rats in TF reflex test.

Pain is a complex experience that incorporates both sensory and affective dimensions. As mention above, intradermal injection of diluted formalin produces both conditioned place aversion (CPA) and a two-phase nociceptive behavioral response in rats. In the present study, we observed that OVX produced a significant facilitation of formalin nociceptive behaviors during phase 2 and a decreased F-CPA score, implying that ovarian hormones in adult female rats may be not only involved in pain sensation but also in pain-related negative emotion. It was reported that subcutaneous injection of 17β-estradiol (E2) induced rat conditioned taste aversion in both sexes [42]. Our previous study further demonstrated that microinjection of E2 into the bilateral rostral anterior cingulate cortex (rACC) induced CPA by upregulating NMDA receptor function and blockade of estrogen receptors by ICI 182,780 prevented F-CPA [21]. The present study further demonstrated that depletion of ovarian hormones markedly suppressed aversive emotion evoked by formalin noxious stimulation. Differently, OVX did not influence foot-shock-induced CPA (S-CPA), a fear-conditioning task. Thus, our present results that OVX suppressed F-CPA but did not affect S-CPA suggest a particular role of ovarian hormones in pain-related aversive learning but not in fear learning processes.

### Ovariectomy and depression

The role of ovarian hormones on the regulation of affective disorders has been established particularly in vulnerable women [43] In fact, the higher incidence and in some cases, severity of depression is associated with the presence or absence of ovarian hormones. Three to five times incidence of major depression is reported in perimenopause than that in other periods of reproductive life [44]. Depressive-like behaviors in rodents, as a consequence of ovarian hormone withdrawal, have also been reported. For instance, in forced swim test and tail suspension test, an increase in immobility time and a decreased in active behaviors those were thought to be indicative of depressive-like behaviors have been observed 2–4 weeks after OVX in both rats and mice, and substitution treatment with E2 partially attenuated these parameters [16], [17], [45], [46]. The present results showed decreased exploratory and active behaviors in open-field test (OFT) and increased immobile behavior in FST at 5 weeks after OVX, and E2 replacement significantly retrieved these behavior disorders. These data confirmed the role of ovarian hormones, especially estrogen, in the regulation of depressive-like affective disorders.

With regard to the development of depressive-like behaviors, there were different reports depending on animal species/strains, behavioral test paradigms and test periods. Estrada-Camarena et al. reported that the immobile behavior was observed only at 1 week but not at 3 and 12 weeks after OVX in female Wistar rats [30]. Also, another study in female Wistar rats showed that the immobile behavior in FST 15 months after OVX did not differ from sham-OVX [47]. In adult female Long-Evans rats, anxiety- and depressive-like behaviors were observed 6 months after OVX [15]. In the current study, OVX-induced depressive-like behaviors were observed at the 5^th^ week after surgery in adult Sprague Dawley female rats, enriching previous published studies.

### Ovariectomy and learning memory

E2 has been reported to promote the formation of new dendritic spines and excitatory synapses in the hippocampus [48]–[50]. E2 also increases hippocampus synaptic strength [51]. Our recent study observed similar changes in the cingulate cortex [21]. These results suggest that hippocampus- or cortex-dependent learning and memories are associated with ovarian hormones. Actually, in humans [52], [53] and animals [54], [55], long-term ovarian hormone loss following surgical menopause has been reported to impair cognition and learning memories. Morris water maze is an experimental method commonly used to evaluate spatial learning and memory in animal models [56]. Despite there were some studies showed an impaired spatial performance in OVX rats and an improved efficiency of spatial learning performance by E2 therapy [57], [58], we did not observed spatial ability deficit in OVX rats by Morris water maze test in the present study. Consistently, Herlitz et al. showed that there were no considerable differences in cognitive performance between premenopausal and postmenopausal women [59]. A possible explanation for this discrepancy is that the cognitive impairment after OVX may be delayed for a longer period of time. A previous study from Markowska and Savonenko [55] indicated that after OVX the cognitive impairment was gradual (taking several months to be detected), initially occurred in tasks that placed more demands on working memory, and then was detected progressively in the easier tasks. A deficit first occurred 4 months after OVX in working memory, while even up to 9 months no differences in spatial reference memory were observed [55]. In our current study, OVX and intact or sham-OVX rats had similar spatial ability in Morris water maze task at the 5^th^ week after surgery. In support of these, a decreased synaptic strength at the hippocampal CA3-CA1 synapses was found only in long-term ovarian hormone loss rats (5 months after OVX) but did not in short-term OVX (7–10 days), suggesting OVX-induced hippocampus-dependent learning and memory deficits might be delayed [60].

**Figure 6:**
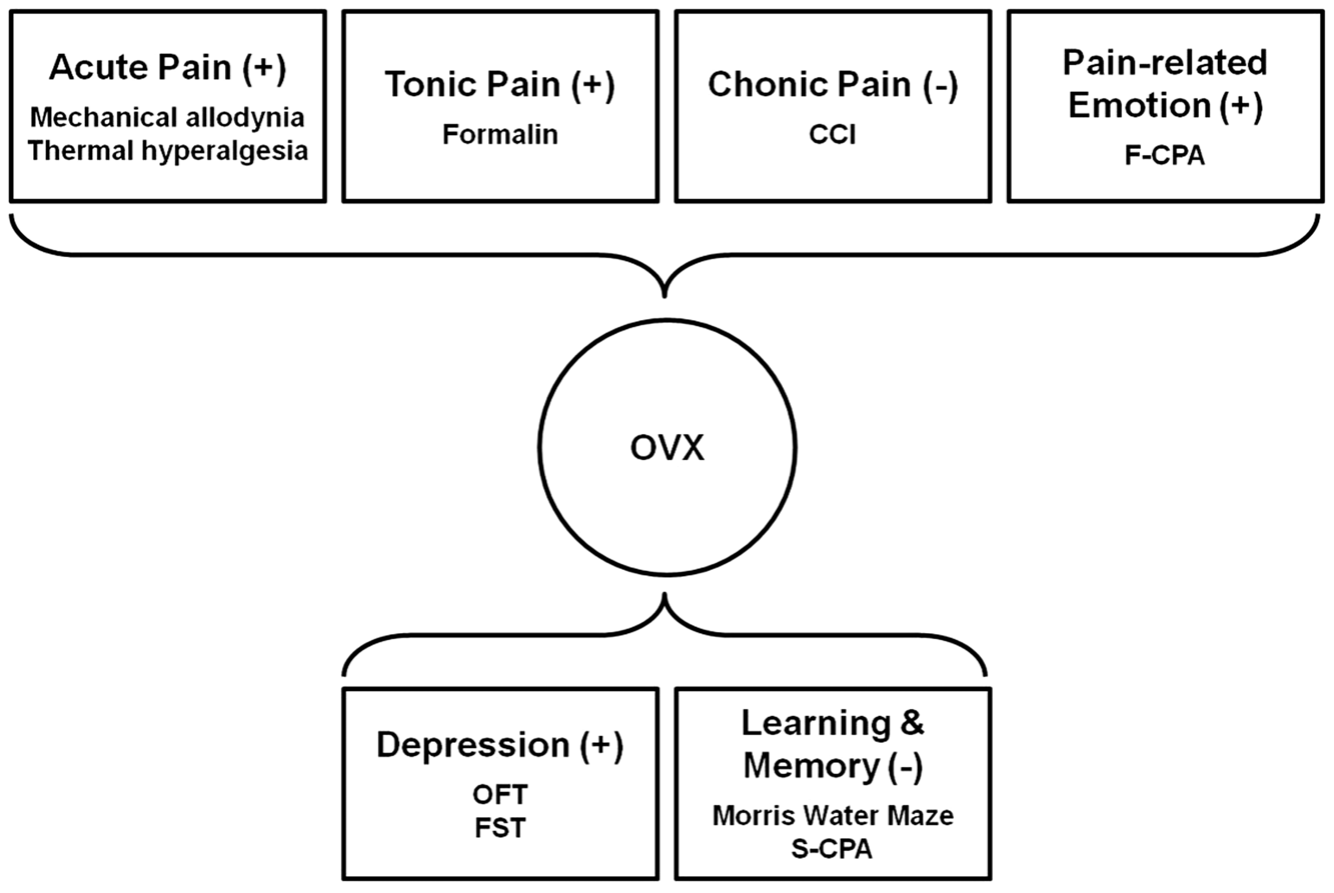
Schematic illustration for effects of OVX on variable behaviors. OVX facilitated acute pain and formalin tonic pain, attenuated pain-related negative emotion and produced depressive-like behaviors, but did not affect spatial ability and fear-related learning and memory.

The present study highlights the complexity of gonadal hormonal influences on brain functions in females. Up to 5 weeks, although OVX rats did not show detectable deficit in spatial ability and contextual fear memory, significant nociceptive facilitation and depressive-like behaviors were developed (Fig. 6).
